# Malaria resurgence risk in southern Europe: climate assessment in an historically endemic area of rice fields at the Mediterranean shore of Spain

**DOI:** 10.1186/1475-2875-9-221

**Published:** 2010-07-31

**Authors:** Sandra Sainz-Elipe, Jose Manuel Latorre, Raul Escosa, Montserrat Masià, Marius Vicent Fuentes, Santiago Mas-Coma, Maria Dolores Bargues

**Affiliations:** 1 Departamento de Parasitología, Facultad de Farmacia, Universidad de Valencia, Av. Vicent Andrés Estellés s/n, 46100 Burjassot, Valencia, Spain; 2 Consorci de Serveis Agroambientals de les Comarques del Baix Ebre i Montsia (CODE), Av. I. Soriano-Montagut 86, 43870 Amposta, Tarragona, Spain

## Abstract

**Background:**

International travel and immigration have been related with an increase of imported malaria cases. This fact and climate change, prolonging the period favouring vector development, require an analysis of the malaria transmission resurgence risk in areas of southern Europe. Such a study is made for the first time in Spain. The Ebro Delta historically endemic area was selected due to its rice field landscape, the presence of only one vector, *Anopheles atroparvus*, with densities similar to those it presented when malaria was present, in a situation which pronouncedly differs from already assessed potential resurgence areas in other Mediterranean countries, such as France and Italy, where many different *Anopheles *species coexist and a different vector species dominates.

**Methods:**

The transmission risk was assessed analysing: 1) climate diagrams including the minimum temperature for *Plasmodium falciparum *and *Plasmodium vivax *development; 2) monthly evolution of the Gradient Model Risk (GMR) index, specifying transmission risk period and number of potential *Plasmodium *generations; 3) ecological characteristics using remote sensing images with the Eurasia Land Cover characteristics database and the monthly evolution of the Normalized Difference Vegetation Index (NDVI); 4) evaluation of *A. atroparvus *population dynamics.

**Results:**

Climatological analyses and GMR index show that a transmission risk presently exists, lasting from May until September for *P. falciparum*, and from May until October for *P. vivax*. The GMR index shows that the temperature increase does not actually mean a transmission risk increase if accompanied by a precipitation decrease reducing the number of parasite generations and transmission period. Nevertheless, this limitation is offset by the artificial flooding of the rice fields. Maximum NDVI values and *A. atroparvus *maximum abundance correspond to months with maximum growth of the rice fields.

**Conclusions:**

The Ebro Delta presents the ecological characteristics that favour transmission. The temperature increase has favoured a widening of the monthly potential transmission window with respect to when malaria was endemic. The combined application of modified climate diagrams and GMR index, together with spatial characterization conforms a useful tool for assessing potential areas at risk of malaria resurgence. NDVI is a good marker when dealing with a rice field area.

## Background

Change of climate factors and other variables related to environmental modifications included within the broad term of global change have a proven impact on the transmission of infectious diseases [1,2], caused by different types of infectious organisms including microparasites (viruses, bacteria, rickettsia and protozoans) and also, as very recently proved, metazoan macroparasites (helminths) [3-5], among which mainly vector-borne diseases [6-9], such as malaria [10-13]. Prediction studies indicate that the incidence of infectious diseases is likely to increase as a consequence of climate change and, in the case of malaria, it has been calculated that 50 million new cases will occur by 2100 [14]. Global warming has been considered as a potential risk for malaria resurgence in northern hemisphere areas [12,15].

Southern Europe is among the most risky regions for malaria resurgence due to (i) the appropriate latitudes with benign climate such as mainly those under mildering influences of the Mediterranean sea, (ii) the closeness to Africa enabling an uncontrolled entry of immigrants from sub-Saharan Africa by means of boats, whether directly through the Mediterranean or indirectly through the Pacific into first the Canary Islands and afterwards the Iberian Peninsula, (iii) the wide presence of anopheline vectors which played an important transmission role during the first half of last century, (iv) the pronounced antropogenic modifications of the environment related to modern development, industry and construction growing giving rise to settlement concentrations consequence of increasing population and tourism which attract a high number of poor immigrants coming from endemic countries and looking for jobs created within the construction and services sectors, and (v) the high immigration rates occurred in recent years mainly from poor endemic areas of developing countries of other continents. All this applies to Spain, where the increase of international travel and immigration from presently malaria-endemic countries has been an exponential phenomenon and global warming has become very evident in the last two decades. In this country, as well as in the neighbouring Portugal, *Anopheles atroparvus *was the most efficient vector [16,17]. At present, this anopheline species shows high population densities similar to those observed in old times when there still were authochthonous transmission areas [18,19]. These facts call for the need to carry out studies on the present potential transmission risk of malaria in areas of Spain, which were historically endemic to assess resurgence probabilities.

In Spain, benign tertian fever caused by *Plasmodium vivax *and, to a lesser degree, malign tertian fever caused by *Plasmodium falciparum *and that of quartan periodicity caused by *Plasmodium malariae*, were endemic until mid last century. In 1943, around 400,000 cases were diagnosed and 1,307 deaths due to malaria were registered. The last autochthonous case was registered in May 1961, and Spain was declared a malaria-free country since it received the official certificate of eradication in 1964 [20]. However, imported cases, mainly stemming from immigrants and tourists, are yearly reported. The surge in travel to exotic countries where malaria is present as well as the increased rate of immigration from endemic countries have led to a steady increase in imported malaria cases from 21 in 1967 to 263 in 1995 and 351 in 2004 [21,22]. Thus, the possibility that Spanish *Anopheles *could facilitate the re-introduction of *Plasmodium *into the country carried by immigrants or travellers who did not follow the appropriate prophylactic measures, has to be taken into account, depending on the receptivity towards its various strains. Currently, new studies are being carried out on the receptivity of the European vector *A. atroparvus *towards African *Plasmodium *strains. Previous studies already revealed that it is not susceptible to the afro-tropical *P. falciparum *strains [6,18,23-26], but susceptible to the European strains [6,27,28], although probably fully susceptible to infection by *P. vivax *strains imported from Africa [29]. In addition, Asian or American *P. falciparum *strains may also be imported [29]. Hence, a transmission resurgence risk, as reflected by the detection of possible autochthonous cases in south European countries as Spain [29], Greece [30,31], Italy [32-34] and France [35,36], and even more northward as in Germany [37], should be taken into account. These facts make it necessary to carry out studies to analyse the present situation and to assess whether the ecological, entomological and social characteristics of the historically endemic areas in Spain allow for the transmission of the disease nowadays. Studies with similar objectives have very recently been carried out in other European countries such as Italy [33,38], France [39-43], Portugal [17] and Germany [44].

**Figure 1:**
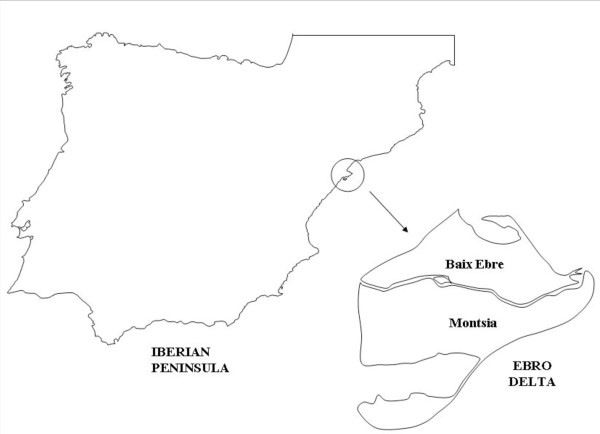
**Map of the study area, the Ebro Delta, Spain**.

A field analysis of potential risk factors of malaria transmission resurgence has so far never been made in Spain. A historically endemic area as the Ebro Delta, at the Mediterranean shore (Figure 1), appears to be the best way for such a study. Malaria in the Ebro Delta was first reported in Tortosa in 1738 and between 1800 and 1900 the disease was included among the priority treatment campaigns prescribed by local physicians [45]. The problem of malaria in the area began to worsen from 1860 onwards after the introduction of rice cultivation [46]. The rapid expansion of this crop together with the bad planning of drainage systems, the precarious conditions of day labourers and deficient hygienic conditions caused an increase of the disease and turned it into a serious health and socioeconomic problem, as a consequence of the decrease of day labourers and the costs incurred by treatments and anti-malaria campaigns. Old data showed that 26,557 out of 70,540 inhabitants of the Ebro Delta were exposed to a paludic environment and that the number of infected people rose from 6,207 in 1912 to 12,545 in 1915, reaching an outstanding 95% in some municipalities of the area [47]. *Plasmodium falciparum *and *P. vivax *were the causes of two thirds of all cases [45]. There has been no indication of malaria transmission since its eradication. Thus, the Ebro Delta presents nowadays a situation of anophelism without malaria, with *A. atroparvus *as the only anopheline species present [16]. This feature shall be highlighted when compared to old malaria endemic areas and presently potential resurgence areas in other Mediterranean countries as France and Italy, where many different *Anopheles *species coexist in the same area and a different vector species dominate, as *Anopheles hyrcanus *in France [39,40] and *Anopheles labranchiae *in Italy [33,34,38]. *Anopheles atroparvus *is an halophilous species following seasonal population dynamics nearly throughout Europe. Changes in climatic conditions, including temperature, evapotranspiration and surface runoff, all key factors to determining mosquito abundance and survivorship [48], may increase the favourable period for the development of the vector and the transmission of the disease.

With the aim to assess whether the present ecological characteristics of the Ebro Delta permit malaria transmission nowadays, the *A. atroparvus *population has been monitored and climatic as well as terrain conditions have been characterized to determine if this parasite/vector association could satisfactorily complete its biological cycle. This assessment has been carried out by means of a climatic analysis and applying the Gradient Model Risk Index based on climatic conditions favouring the development of the parasite and vector [49,50]. For terrain analysis, satellite imagery, known to furnish valuable data for the forecast of malaria [51], has been used by means of remote sensing information selected to characterize the overall land cover of rice fields of the Ebro Delta as data from the Eurasian Land Cover database [52], and the Normalized Difference Vegetation Index (NDVI) which has been proved to be highly efficient for prediction studies in water-related parasitic diseases [53].

## Methods

### Study area

The Ebro Delta is a Mediterranean ecosystem, situated in the province of Tarragona, Spain, covering 24,000 ha (Figure 1), of which 8,000 ha form a Natural Park. The delta plain, at sea level, is divided by the Ebro river into two hemideltas. The left part includes the municipality of Deltebre, Baix Ebre zone, and the right hemidelta includes the municipalities of Amposta, Sant Jaume d'Enveja and Sant Carles de la Rapita, Montsia zone.

Since the construction of the irrigation canals, on the right hemidelta in 1860 and on the left in 1912, rice cultivation has taken up a great part of the delta plain, currently around 65%. The dominance of this crop, characterized by extensive water networks throughout the entire delta, constitutes a favourable environment for various culicid species, including *A. atroparvus*.

### Climatic features

The climatic features of the Ebro Delta have been analysed to determine whether they permit the transmission of *Plasmodium*. This characterization was carried out based on climate data corresponding to a 26-year-period (1961-1986), obtained from the weather station situated in Tortosa, belonging to FAO. The following climate data were analysed (Table 1): mean maximum temperature (MMT), mean minimum temperature (MmT), mean environmental temperature (MET), all in °C, precipitation in mm (Pt), relative humidity in % (RH), potential evapotranspiration in mm (PET), wind speed in m/s (WM), vapor pressure in hPa (VP), and global radiation in W/m^2 ^(GR).

**Table 1:** Climate data of the 26-year-period analysed, retrieved from Tortosa (Ebro Delta, Spain) weather station.

	Jan	Feb	Mar	Apr	May	Jun	Jul	Aug	Sep	Oct	Nov	Dec	Year
MMT (°C)	13.5	15.5	18.1	20.6	23.4	27.3	30.2	30.0	27.6	17.8	14.0	22.6	21.7
MmT (°C)	4.8	5.6	7.6	10.1	13.2	17.2	19.8	19.9	17.7	12.9	8.9	6.1	12.0
MET (°C)	9.2	10.6	12.9	15.4	18.3	22.3	25.0	25.0	22.7	17.8	13.4	10.1	16.9
Pt (mm)	29.0	26.0	40.0	45.0	68.0	47.0	20.0	38.0	84.0	72.0	43.0	53.0	565.0
RH (%)	66.0	63.0	65.0	59.0	63.0	61.0	60.0	65.0	64.0	69.0	67.0	68.0	64.0
PET (mm)	39.0	53.0	78.0	113.0	136.0	150.0	182.0	157.0	112.0	70.0	45.0	55.0	99.0
GR (W/m^2^)	172.0	241.0	298.0	418.0	474.0	527.0	552.0	482.0	358.0	256.0	190.0	149.0	343.0
WM (m/s)	3.9	3.8	3.5	3.7	3.4	2.6	3.4	3.3	3.1	3.0	3.5	3.9	3.4
VP (hPa)	7.7	8.0	9.6	10.4	13.2	16.5	19.0	20.6	17.6	14.0	10.3	8.4	12.9

MMT = mean maximum temperature; MmT = mean minimum temperature; MET = mean environmental temperature; Pt = precipitation; RH = relative humidity; PET = potential evapotranspiration; GR = global radiation; WM = wind speed; VP = vapor pressure.

A climate diagram for the area has been created, based on proposed models [54] and according to methods used previously [55], with emphasis on the duration and periodicity of humid and dry intervals along the study period. Also, climate diagrams for the two study years of 2005 and 2006 have been created to determine possible variations in duration and periodicity of humid and dry periods caused by the increasing temperatures of recent years. Precipitation data (mm) and mean environmental temperature (°C), used when creating the aforementioned climate diagrams, were obtained from the weather station of the Servei Meteorologic de Catalunya, located in Amposta.

### Terrain characterization by remote sensing data

The unchangeable patterns of rice sowing and harvesting along the last decades and the crop's predominance in the Ebro Delta justify the use of satellite images (although not exactly corresponding to the two-year field study period) for an appropriate characterization of this area, thus determining whether its features allow for the completion of the vector cycle.

To study related remote sensing data, a total of 72 10-day (dekad) composite images from the Advanced Very High Resolution Radiometer (AVHRR) sensor on board the National Oceanic and Atmospheric Administration (NOAA) environmental satellite, have been analysed. This dataset archive comprises the period April 1992 to March 1993 and February 1995 to January 1996 and was downloaded from the Global Land 1-km project Internet site. Particularly, the monthly evolution of corresponding NDVI values has been obtained to show the general response of vegetation to water availability (rainfall) and in this case of rice growing to man-made irrigation. This index produces values in the range of -1.0 to 1.0, where increasing positive values indicate increasing green vegetation and lower values indicate non-vegetated surface features such as water, barren areas, ice and snow or clouds [53].

Also, the Eurasia Land Cover characteristics database using 1-km AVHRR data covering the period from April 1992 to March 1993 [52] has been analysed to determine the majority classes of each of the classifications included in this database.

All images were geo-referenced and processed using Erdas Imagine 8.3.1 software. ArcView GIS 3.2a software and its Spatial Analyst application were used to obtain values corresponding to surfaces occupied by each class of NDVI and Land Cover for each of the three polygons created, namely the entire Ebro Delta, the left hemidelta (Baix Ebre zone) and the right hemidelta (Montsia zone). Of the obtained values, those related to the majority value in the case of Land Cover and to the average in the case of NDVI have been considered.

### Entomological study

**Figure 2:**
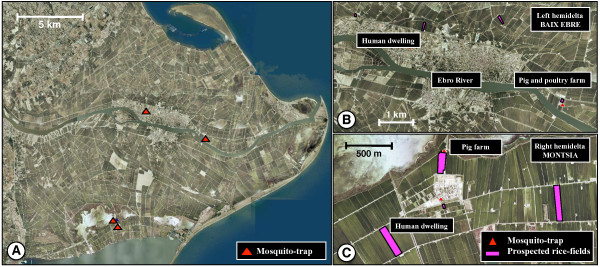
**Orthophotograph 1:25000 of Ebro Delta, Spain**. General view (A); mosquito traps and prospected rice-fields in left hemidelta (Baix Ebre) (B) and right hemidelta (Monsia) (C).

The entomological study has been performed from May to October 2005 by means of bimonthly surveys. Adult and larval stages of the various mosquito species were captured using CDC-light traps, following the methodology detailed in other recent studies [34,40] and installed at four sites on either side of the Ebro Delta: two in the Baix Ebre zone, one on a pig and poultry farm, one situated in a human dwelling in the town centre of Deltebre; two in the Montsia zone, one on a pig farm and one in a human dwelling situated in the centre of the town of Poble Nou (Figure 2). To obtain larval stages eight rice fields were chosen, four in the Baix Ebre zone and four in the Montsia zone, where non-permanent water spots were surveyed at random. Five samples were taken at different spots along the margins of each rice field surveyed. Collected specimens were classified at species level by means of their morphological characteristics and descriptions of local populations and keys [56,57]. In the case of larval stages, differentiation was carried out considering 1-2 larvae, 3-4 larvae, nymphs and eggs. Species and strain belonging was previously verified by sequencing of selected markers of the nuclear ribosomal DNA by the dideoxy chain-termination method [16,58]. Present population dynamics of *A. atroparvus *encountered in the study area was analysed by means of this entomological study.

### Transmission risk

The potential malaria transmission risk in the Ebro Delta was analysed by means of:

A) including the minimum temperature required for the development of *Plasmodium *species in the aforementioned climate diagrams to analyse which months present favourable conditions for the development of the parasite;

B) calculating the Gradient Model Risk Index (GMR index) applied to forecast the malaria transmission risk [49,50], i.e. the monthly evolution of accumulated values of the index was analysed to gain insight into possible transmission periods along the year; this index considers only climatic parameters as the minimum mean temperature required for the development of the parasite inside the vector, precipitation (mm) and PET (mm) per month, calculated by the equation:

where GDD is growing degree-days, R is rainfall, and PET is potential evapotranspiration.

The GMR index shows that a transmission risk exists when its value equals 116, the value required for one *Plasmodium *generation, or is higher. Minimum temperatures required for the development of *Plasmodium *species are: 15°C for *P. vivax *and 18°C for *P. falciparum *[7,15,59-61].

## Results

### Climatic characterization

**Figure 3:**
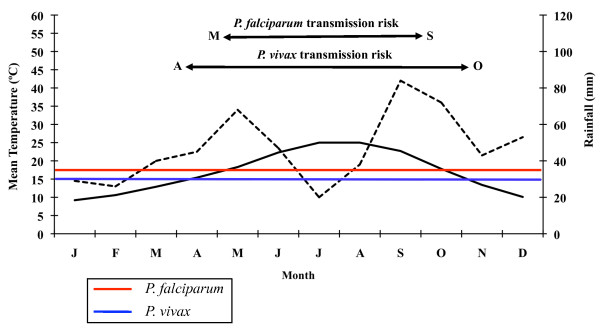
**Ebro Delta climate diagram for a 26-year-period**. Average temperatures (solid line) and precipitations (broken line). Horizontal blue and red lines indicate minimal temperature required for *P. vivax *(15°C) and *P. falciparum *(18°C) development respectively. Data from Tortosa, Tarragona, Spain, weather station.

**Figure 4:**
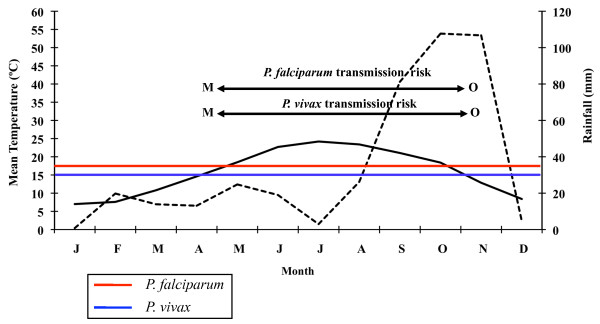
**Ebro Delta climate diagram for 2005**. Average temperatures (solid line) and precipitations (broken line). Horizontal blue and red lines indicate minimal temperature required for *P. vivax *(15°C) and *P. falciparum *(18°C) development respectively. Data from Amposta, Tarragona, Spain, weather station.

**Figure 5:**
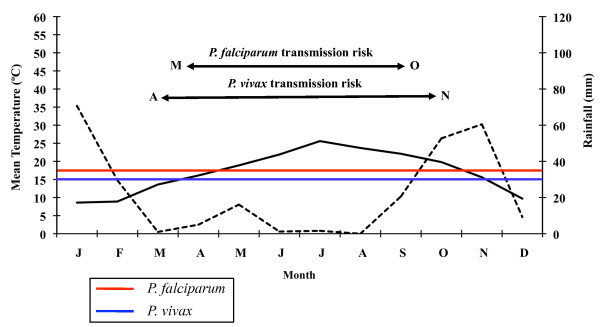
**Ebro Delta climate diagram for 2006**. Average temperatures (solid line) and precipitations (broken line). Horizontal blue and red lines indicate minimal temperature required for *P. vivax *(15°C) and *P. falciparum *(18°C) development respectively. Data from Amposta, Tarragona, Spain, weather station.

The following data shall be emphasized among the results from the climatic analysis of the study area: mean annual temperature of 16.9°C (9.2°C in January and 25.0°C in August); mean annual maximum temperature of 21.7°C (13.5°C in January and 30.2°C in July); mean annual minimum temperature of 12.0°C (4.8°C in January and 19.9°C in August). Accumulated precipitation along the year does not exceed 565 mm. September (84 mm) and October (72 mm) are the rainiest months of the year. Maximum values of potential evaporation are reached in April and September, reaching its peak in July (182 mm) and relative humidity is around 60% in all months of the year, bar April. The wet period is situated between September and June according to the climate diagram created for 26 consecutive years (Figure 3). The dry period lasts from July until August. The climate diagrams corresponding to 2005 and 2006 (Figures 4 and 5) show, when compared to the 1961-1986 period, an increase of the duration of the dry period, which in either year began in March and lasted until August 2005 and September 2006, respectively. The wet period of the two study years is divided into two parts: February and from September until December in 2005; and from January to February and from October until November in 2006.

### Terrain characterization by remote sensing data

**Figure 6:**
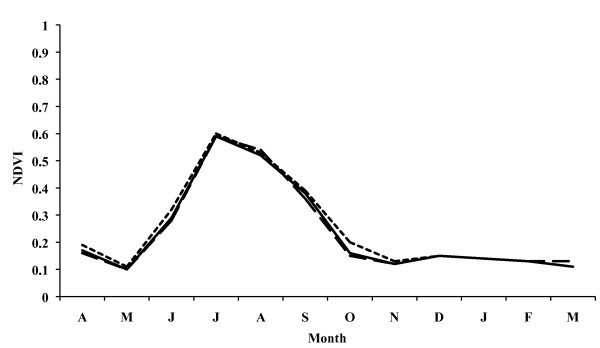
**Monthly evolution of NDVI values in the Ebro Delta from April 1992 to March 1993**. Entire Ebro Delta (continuous line), left hemidelta (dashed line) and right hemidelta (dotted line).

**Figure 7:**
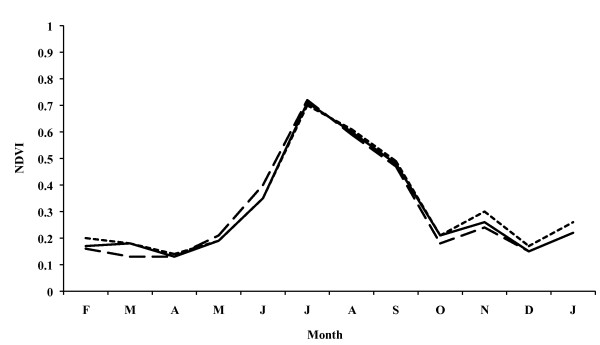
**Monthly evolution of NDVI values in the Ebro Delta from February 1995 to January 1996**. Entire Ebro Delta (continuous line), left hemidelta (dashed line) and right hemidelta (dotted line).

When analysing the terrain features of the Ebro Delta by means of Eurasia Land Cover, it can be seen that irrigation farming, especially rice cultivation, is predominant (Table 2). NDVI values show a progressive increase from May in the two annual periods analyzed, reaching the maximum NDVI value in July (0.6 in the annual period comprising April 1992 to March 1993; 0.7 in the annual period comprising February 1995 to January 1996), reaching their maximum values in the May-October period (Fig. 6 and 7). These results are similar in the three zones considered, namely the entire Delta, the left hemidelta and the right hemidelta.

**Table 2:** Terrain features of the Ebro Delta by means of Eurasia Land Cover

Land Cover Data Base	Entire Ebro Delta	Left Hemidelta	Right Hemidelta
Global ecosystems	Crops, Grass, Shrubs	Crops, Grass, Shrubs	Crops, Grass, Shrubs
IGBP Land Cover Classifications	Cropland/ Natural Vegetation Mosaic	Cropland/ Natural Vegetation Mosaic	Cropland/ Natural Vegetation Mosaic
Land Cover Eurasia Seasonal	Cropland (Wheat)/ Grassland Mosaic	Cropland (Wheat)/ Grassland Mosaic	Cropland (Wheat)/ Grassland Mosaic
Simple Biosphere Model	Agriculture or C3 Grassland	Agriculture or C3 Grassland	Agriculture or C3 Grassland
Simple Biosphere 2 Model	Agriculture or C3 Grassland	Agriculture or C3 Grassland	Agriculture or C3 Grassland
Biosphere-Atmosphere Transfer Scheme	Crops, Mixed Farming	Crops, Mixed Farming	Crops, Mixed Farming
Vegetation Liteforms	Annual Grass Vegetation	Annual Grass Vegetation	Annual Grass Vegetation
U.S. Geological Survey Land Use/Land Cover System	Cropland/ Grassland Mosaic	Cropland/ Grassland Mosaic	Cropland/ Grassland Mosaic

Majority classes of the three considered polygons (entire Delta, left hemidelta and right hemidelta) corresponding to the different classifications of the Land Cover Dates Bases.

### Entomological study

**Figure 8:**
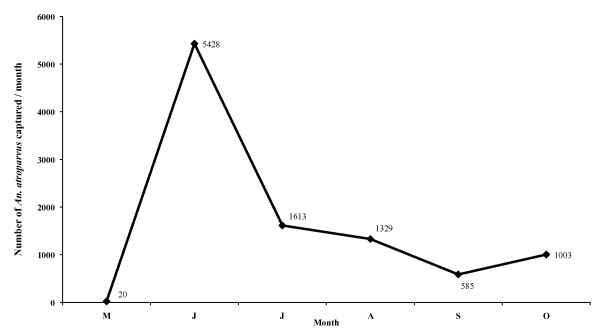
**Monthly evolution of *A. atroparvus *captures during the surveyed year 2005 from May to October**.

Since the beginning of the study in May 2005, a total of 16,130 adult mosquitoes were captured, including 9,978 *A. atroparvus *(61.86%) and 6,152 culicine species (38.14%) belonging to *Culex pipiens, Culex modestus, Ochlerotatus caspius *and *Culiseta longiareolata. Anopheles atroparvus *was detected along the entire study period (May-October). The follow-up of the total number of anophelines captured allowed for the detection of its maximum density in the period from June until August, its decrease in September and a considerable increase in October (Figure 8). The detection of a male *A. atroparvus *specimens during the first survey on 11th May on a pig farm in Poble Nou (Montsia zone) is particularly noteworthy, especially since larval stages neither of anopheline nor of culicine had been detected before in the surrounding rice fields or in any other parts of the Delta.

Concerning larval stages, of the total of 661 culicid larvae collected, 647 (97.88%) of which were *C. pipiens *and *C. modestus*, and only 14 (2.12%) *A. atroparvus*. The larval stage of *A. atroparvus *was only sporadically detected, which made it impossible to carry out a larval stage population study of this species. *Anopheles atroparvus *larvae were first detected during the July rice field survey. However, breading sites were first detected in localities situated away from the rice fields, in the irrigation system belonging to the previously mentioned pig farm and in a puddle.

### Transmission risk

The results obtained from the climate diagrams and the calculation of the GMR index indicate that a potential malaria transmission risk in the Ebro Delta does indeed exist. The climate diagram concerning the 26-year period (Figure 3) shows that the minimum temperature required for the development of the two *Plasmodium *species is situated between May and September for *P. falciparum*, and between April and October for *P. vivax*. In 2005, the favourable transmission period for the two *Plasmodium *species considered was between May and October (Figure 4). Only one year later (2006) this period extended from April to November for *P. vivax *and was the same as in the previous year, namely May to October for *P. falciparum *(Figure 5).

**Figure 9:**
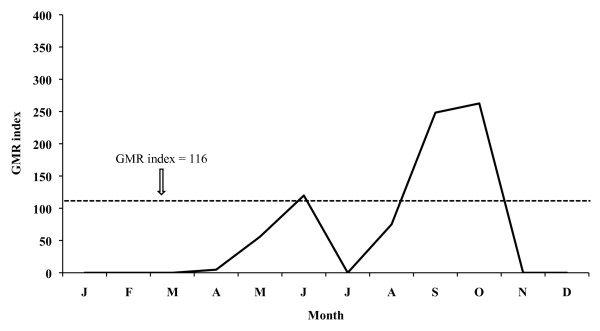
**Monthly evolution of malaria risk transmission for *P. vivax *in the Ebro Delta**. Values obtained by means of Gradient Model Risk Index (GMR index) based on 26 years of weather monitoring.

**Figure 10:**
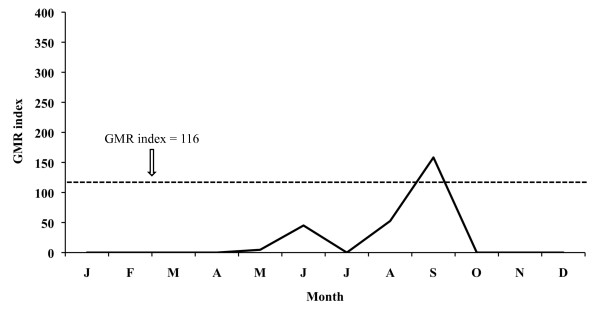
**Monthly evolution of malaria risk transmission for *P. falciparum *in the Ebro Delta**. Values obtained by means of Gradient Model Risk Index (GMR index) based on 26 years of weather monitoring.

The monthly evolution of the GMR index concerning the 26-year period places the potential transmission risk in June, September and October for *P. vivax *(Figure 9), thus allowing for four potential generations, and in September for *P. falciparum *(Figure 10), allowing for two potential generations.

**Figure 11:**
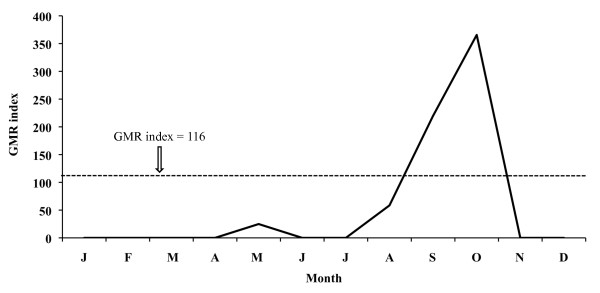
**Monthly evolution of malaria risk transmission for *P. vivax *in the Ebro Delta in 2005**. Values obtained by means of Gradient Model Risk Index (GMR index).

**Figure 12:**
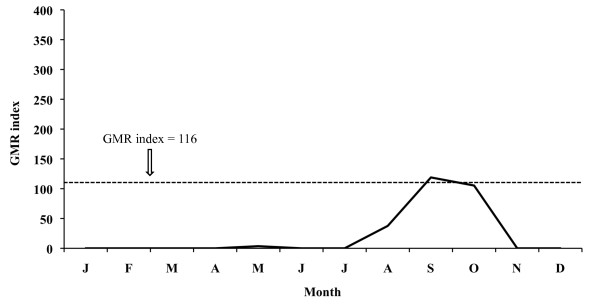
**Monthly evolution of malaria risk transmission for *P. falciparum *in the Ebro Delta in 2005**. Values obtained by means of Gradient Model Risk Index (GMR index).

**Figure 13:**
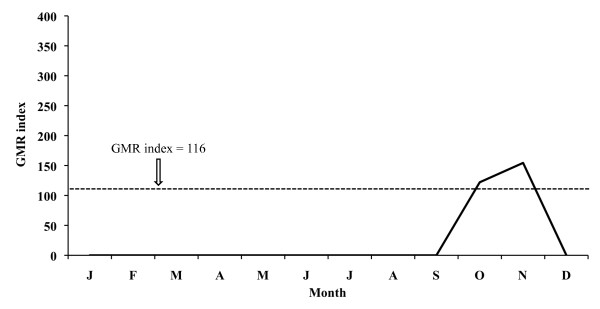
**Monthly evolution of malaria risk transmission for *P. vivax *in the Ebro Delta in 2006**. Values obtained by means of Gradient Model Risk Index (GMR index).

**Figure 14:**
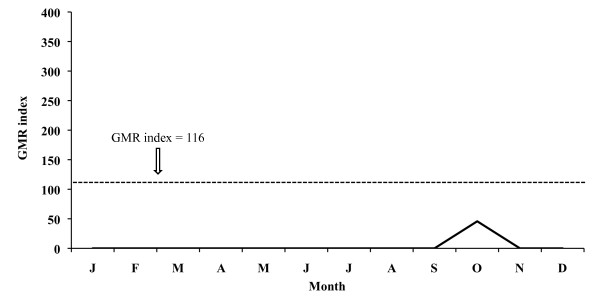
**Monthly evolution of malaria risk transmission for *P. falciparum *in the Ebro Delta in 2006**. Values obtained by means of Gradient Model Risk Index (GMR index).

The aforementioned index for 2005 places the potential transmission risk in September and October for *P. vivax *(Figure 11), thus making the development of four potential generations possible, and in September for *P. falciparum *(Figure 12) allowing for one generation. In 2006 the monthly evolution of the GMR index places the potential transmission risk for *P. vivax *(Figure 13) between October and November. The index, however, does not show any transmission risk for *P. falciparum *(Figure 14) along 2006.

## Discussion

The analysis of the various classifications of the Land Cover database shows that in the Ebro Delta irrigation farming, namely rice, predominates, which is shown at the end of April and the beginning of May and reaches its maximum growth in July and August, respectively. Monthly NDVI values reach their maximums in July and August coinciding with the dry period in the Ebro Delta, reflecting the growth of rice characterized by irrigation through artificial flooding. Thus, the rice fields in the Delta provide an ideal environment for the development and breeding of *A. atroparvus *owing to the existence of great masses of clean, motionless water, as already noted long ago [62,63]. Rice cultivation periods, moreover, coincide with temperatures permitting the completion of the vector cycle, as shown by the detection of *A. atroparvus *during all the months studied. Mosquito maximum abundance in June, July and August also coincides with NDVI maximum values as well as with maximum growth in the rice fields. The importance of cultures of this kind of crop in the development of malaria vectors has already been noted in other south European countries [33,38,42,64,65].

A potential malaria transmission risk in the Ebro Delta is revealed when considering the climate data and the characterization of the study area along the 26-year period analysed. The climate diagram indicates that favourable temperature conditions can be found between May and September for *A. atroparvus *and *P. falciparum*, and such a favourable period extends from April until October for *P. vivax*. The results of the GMR index place the transmission risk solely in June, September and October for *P. vivax *and in September for *P. falciparum*. The calculation of this index makes it possible to define the period of potential transmission risk, considering climatic factors only, but in a much more precise manner, taking also into account the minimum temperature required for parasite and vector development as well as the presence of rain water needed for the completion of the *A. atroparvus *life cycle. Nonetheless, the insufficiency of water needed for vector development in the Ebro Delta, typical for the dry period between June and August, is compensated by the artificial flooding of the rice fields. Therefore, the transmission risk period should be extended to May and September for *P. falciparum *and to May and October for *P. vivax*.

Relative humidity is another essential factor in malaria transmission. Although *Plasmodium *parasites are not affected by relative humidity, anopheline mosquito activity and survival are. If the average monthly relative humidity is below 60%, it is believed that the lifespan of the mosquito is shortened to such an extent that malaria transmission does not occur [66]. The climatic analysis of the study area shows that relative humidity is above 60% along the entire year, bar April. Thus, suitable conditions for the development of *A. atroparvus *and *Plasmodium *turn the Ebro Delta into an ecosystem with favourable climatic characteristics for malaria transmission.

The results obtained agree with old observations [45], which noted that 99% of all new malaria cases in the Delta were detected at the end of summer, that the first cases tended to appear in July, and that epidemics occurred in August, particularly during the last three weeks of that month with the detection of numerous cases. Our results reveal that the favourable transmission period is currently longer than it was, starting two months before, in May, and lasting until September in the case of *P. falciparum *and until October in case of *P. vivax*, respectively. The discovery of larvae in a different place from a rice field (the typical breeding site of *A. atroparvus *in the Ebro Delta) and the discovery of males in May (up until then no larval stages of Anophelinae and Culicinae had been detected in the rice-fields of the Delta) support the prolongation of the malaria transmission risk until October, although the rice cultivation period is completed by then.

Climate data for 2005 and 2006 show that although an increase of temperatures took place, favouring an extension of the transmission period, at the same time a decrease in precipitation occurs, resulting in an extension of the dry period, which in turn would a priori negatively affect the favourable period for vector development if artificial irrigation water would not be available. Population dynamics of *A. atroparvus *populations in the Ebro Delta follow a typical thermophilic trend where rice fields ensure a constant presence of water. The highest values were recorded in the period from June until August, when the abundance of the population peaked. Lowest values were recorded in September, but a new peak appeared in October-November. This seasonal dynamics is quite similar to those presented by *A. labranchiae *in Italy [34] and *A. hyrcanus *in France [40], both assessed during the same biannual period (2005-2006).

Transmission risk analysis for 2005 indicates that conditions for vector development of the two *Plasmodium *species considered are still suitable in the May-September period for *P. falciparum *and in May-October for *P. vivax*. Although the GMR index shows, in the case of *P. vivax*, a disappearance of the transmission risk in June, it has to be kept in mind that the rice field irrigation counteracts the lack of rain water in that month and consequently the transmission risk persists.

Climate data analysis for 2006 confirms that the increase in temperatures prolongs the favourable period for both vector and *P. vivax *development, lasting from May until November. Although the climate diagram shows that the risk period starts in April (favourable temperatures), results derived from the GMR index and the fact that rice is not grown in that month, suitable conditions for the completion of the *Anopheles *life cycle do not exist, and therefore April can not be considered within the potential risk period. However, the GMR index did not indicate a *P. falciparum *transmission risk in 2006 as a consequence of reduced precipitation in that year. Thus, *P. falciparum *sees its favourable period limited to May until September, when the rice field irrigation counteracts the lack of rain water required for the completion of the *Anopheles *life cycle, and temperatures, following the climate diagram, facilitate parasite and vector development.

## Conclusions

The evolution of the GMR index shows that the increase in temperatures does not actually mean an increase in the malaria transmission risk if this is accompanied by a decrease in precipitations. Although temperatures favour parasite development, the lack of water prevents vector development. This fact reduces the number of parasite generations as well as the transmission period. Nevertheless, this limitation is offset by the artificial flooding of the rice fields in the Delta. Therefore, the use of this index, which solely considers the existence of breeding sites and periods as a consequence of precipitation, has to be combined with a spatial characterization of the study area, which helps to detect breeding sites and periods. NDVI from remote sensing sources proves to be a good marker for such a purpose when dealing with a rice field area.

The results obtained make it possible to conclude that the Ebro Delta currently presents ecologically favourable characteristics for the re-appearance of malaria if an appropriate malaria strain would be introduced and that the month window period for potential transmission has even widened due to present global warming impact when compared to last century when this area was endemic. This fact makes it necessary to verify whether the *Plasmodium *strains potentially introducible by infected immigrants, tourists or travellers arriving to Spain could be compatible with the European *A. atroparvus*. Additionally, factors that might influence vector population dynamics as well as the transmission of *Plasmodium *(blood preferences of the vector, aggressiveness, gonotrophic cycle, etc.) have, therefore, to be analysed in the Ebro Delta area in question.

## Competing interests

The authors declare that they have no competing interests.

## Authors' contributions

SSE and JML gathered and analyzed the data. SSE carried out the spatial analysis. RE and MM contributed in the field study design and analysis. MV participated in the forecast-index analysis. SMC and MDB devised, designed and coordinated the study, and wrote the manuscript. All authors read and approved the final manuscript.
